# 1-Phenyl-6,7,8,9-hexa­hydro-1*H*,5*H*-cyclo­hepta­[1′,2′:2,3]pyrido[6,5-*c*]pyrazol-4-amine: a new tacrine analogue

**DOI:** 10.1107/S1600536808013366

**Published:** 2008-05-10

**Authors:** Lijun Zhang, Daxin Shi, Jiarong Li, Ling Zhang, Yanqiu Fan

**Affiliations:** a School of Chemical Engineering and the Environment, Beijing Institute of Technology, Beijing 100081, People’s Republic of China

## Abstract

The title compound, C_17_H_18_N_4_, contains a pyrazolopyridine system fused with a seven-membered carbocyclic ring. The pyrazole ring is coplanar with the pyridine ring, while the phenyl ring is twisted by a dihedral angle of 14.38 (14)° with respect to the pyridine ring. The seven-membered ring displays a chair conformation. The packing is stabilized by N—H⋯N hydrogen bonds and N—H⋯π(arene) inter­actions.

## Related literature

For related literature, see: Gracon *et al.* (1998 ▶); Haviv *et al.* (2005 ▶); Kelley *et al.* (1988 ▶); Kim *et al.* (1996 ▶); Lin *et al.* (2007 ▶); Stachlewitz *et al.* (1997 ▶); Zocchi *et al.* (1996 ▶); Erast *et al.* (1987 ▶).
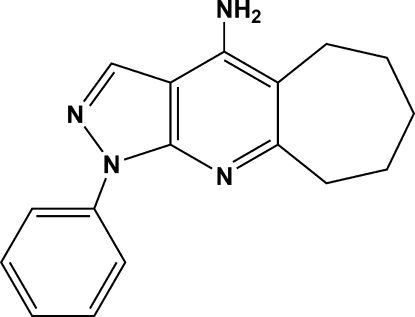


## Experimental

### 

#### Crystal data

C_17_H_18_N_4_
*M* *_r_* = 278.35
Monoclinic, 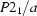

*a* = 13.694 (13) Å
*b* = 6.888 (6) Å
*c* = 16.929 (16) Å
β = 112.417 (12)°
*V* = 1476 (2) Å^3^
*Z* = 4
Mo *K*α radiation
μ = 0.08 mm^−1^
*T* = 293 (2) K
0.24 × 0.18 × 0.10 mm

#### Data collection

Rigaku Saturn diffractometer
Absorption correction: multi-scan (Jacobson, 1998 ▶) *T*_min_ = 0.982, *T*_max_ = 0.992
10708 measured reflections
2593 independent reflections
2004 reflections with *I* > 2σ(*I*)
*R*_int_ = 0.045

#### Refinement

*R*[*F* ^2^ > 2σ(*F* ^2^)] = 0.064
*wR*(*F* ^2^) = 0.162
*S* = 1.13
2593 reflections
190 parameters
H-atom parameters constrained
Δρ_max_ = 0.13 e Å^−3^
Δρ_min_ = −0.16 e Å^−3^

### 

Data collection: *CrystalClear* (Rigaku, 2004 ▶); cell refinement: *CrystalClear*; data reduction: *CrystalClear*; program(s) used to solve structure: *SHELXS97* (Sheldrick, 2008 ▶); program(s) used to refine structure: *SHELXL97* (Sheldrick, 2008 ▶); molecular graphics: *ORTEPIII* (Burnett & Johnson, 1996 ▶), *ORTEP-3 for Windows* (Farrugia, 1997 ▶) and *PLATON* (Spek, 2003 ▶); software used to prepare material for publication: *SHELXL97*.

## Supplementary Material

Crystal structure: contains datablocks I, global. DOI: 10.1107/S1600536808013366/dn2339sup1.cif

Structure factors: contains datablocks I. DOI: 10.1107/S1600536808013366/dn2339Isup2.hkl

Additional supplementary materials:  crystallographic information; 3D view; checkCIF report

## Figures and Tables

**Table 1:** Hydrogen-bond geometry (Å, °)

*D*—H⋯*A*	*D*—H	H⋯*A*	*D*⋯*A*	*D*—H⋯*A*
N1—H1*B*⋯N4^i^	0.86	2.28	3.139 (4)	175
N1—H1*A*⋯*Cg*1^ii^	0.86	2.84	3.608 (4)	150

Symmetry codes: (i) 
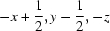
; (ii) 
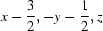
. *Cg*1 is the centroid of the benzene ring.

**Fig. 1.**
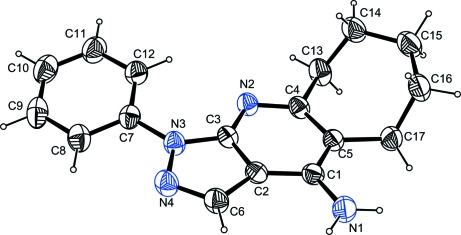
The molecular structure of the title compound, with the atom- labelling scheme. Displacement ellipsoids are drawn at the 30% probability level. H atoms are represented as small spheres of arbitrary radii.

**Fig. 2.**
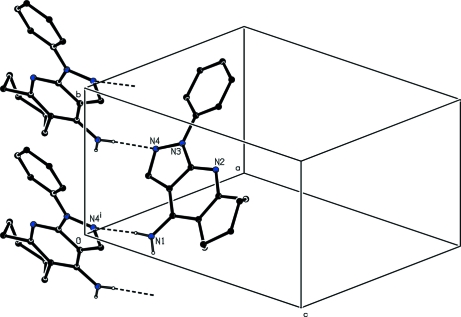
Partial packing view showing as dashed lines the N-H···N hydrogen bondings. H atoms not involved in hydrogen bonds have been omitted for clarity. [Symmetry code: (i) -x+1/2, y-1/2, -z]

**Fig. 3.**
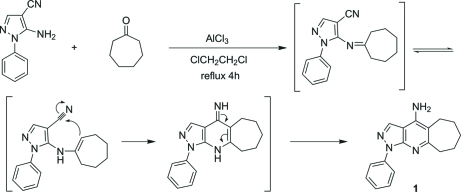
Reaction pathway for the synthesis of the title compound.

## supplementary crystallographic information

### Comment

Tacrine (9-amino-1,2,3,4-tetrahydroacridine, THA) was the first acetylcholinesterase inhibitor approved for the palliative treatment of Alzheimer's disease (AD) (Gracon *et al.*, 1998). But due to its undesirable side effects, especially its hepatotoxicity, the clinical usefulness is limited (Stachlewitz *et al.*, 1997), and the research on seeking new AChE inhibitors with improved activity and reduced adverse side effects are progressing (Haviv *et al.*, 2005). In view of the considerable biological and medicinal activities of pyrazole ring compounds, such as important adenosine antagonist (Zocchi *et al.*, 1996), antifungals (Kim *et al.*, 1996), plant growth regulators (Erast *et al.*, 1987), anti-tumor agents (Lin *et al.*, 2007), anticonvulsant agents (Kelley *et al.*, 1988), *etc*. we designed and synthesized the compound 11-phenyl-1,5,6,7,8,9- hexahydrocyclohepta[*b*]pyrazolo[4,3-*e*]-pyridin-4-amine (a new Tacrine analogue) (Scheme 1).

In the title compound, the fused pyrazolopyridine moiety is roughly coplanar, with an angle of 1.4° between the pyrazole and the pyridine rings (Fig. 1), The largest deviation from the mean plane being 0.014 (2)Å at C5. Both the C3—N2 [1.345 (3) Å] and the C4—N2 [1.348 (3) Å] bond lengths of pyridine are much shorter than those observed in the pyrazole ring [C3—N3 1.378 (3) Å and N3—N4 1.380 (3) Å], indicating higher aromatic nature of the pyridine ring than the pyrazole. The amino group is sligthly twisted by 1.71 (8)° with respect to the pyrazolopyridine moiety The benzene is also twisted and make a dihedral angle of 14.38 (14)\% with the pyrazolopyridine moiety, The seven-membered ring displays a chair conformation.

There are strong intermolecular N—H···N hydrogen bonds between the amino group and one N atom of the pyrazole ring (Table 1, Fig. 2). The packing is further stabilized by N—H···π(benzene) interactions (Table 1).

### Experimental

A solution of 0.2 g of 5-amino-4-cyanopyrazole (1.1 mmol,), 0.16 g of AlCl_3_ (1.2 mmol,) in 5 ml of 1,2-dichloroethane was refluxed for 4 h (monitored by TLC). The reaction mixture was cooled, dispersed into THF/water (2:1 vol.) and titrated to pH=7 by 20% sodium hydroxide. Then, the mixture was stirred for 30 min. and extracted three times with dichloromethane, the organic layers were dried and evaporated at reduced pressure to give the solid product  (Fig. 3). The title compound 1 was purified by silica gel column chromatography eluting with ethyl acetate/light petroleum in 40% yield.

Its single-crystal was cultured from a solution of ethanol by slow evaporation at room temperature.

The product 1, white crystal, m.p. 194–195°C, was characterized by ^1^H NMR, ^13^C NMR, ESI, IR, EA. IR (KBr) (cm^-1^): 3482 and 3350 (NH), 2922 (CH), 1638 and 1594 (C=N), 1501, 1358; ^1^H NMR (400 MHz, CDCl_3_, δp.p.m.): 1.62–1.90 (m, 6H, alkyl-H), 2.66–2.69 (t, 2H, alkyl-H), 3.08–3.01 (t, 2H, alkyl-H), 4.60 (s, 2H, NH_2_), 7.22–7.26 (t, J=7.4 Hz, 1H), 7.46–7.50 (t, J=7.5 Hz, 2H), 7.98 (s, 1H), 8.30–8.32 (d, J=7.6 Hz, 2H); ^13^C NMR (400 MHz, CDCl3, δp.p.m.): 25.40, 26.79, 27.56, 32.16, 39.81, 106.11, 113.24, 120.91 (2 C), 125.37, 128.89 (2 C), 130.61, 140.02, 143.69, 149.52, 165.25; ESI [*M*+H]^+^: 279.1; Anal. Calcd. for C_17_H_18_N_4_: C, 73.35; H, 6.52; N, 20.13. Found: C, 73.17; H, 6.52; N, 19.99.

### Refinement

All H atoms attached to C atoms were fixed geometrically and treated as riding with C—H = 0.93 Å (aromatic) or 0.98 Å (methine) with *U*_iso_(H) = 1.2*U*_eq_(C). H atoms attached to N were located in difference Fourier maps but introduced in calculated positions and treated as riding on the N atoms with N-H = 0.86 Å and *U*_iso_(H) = 1.2*U*_eq_(N).

### Crystal data

**Table:**

C_17_H_18_N_4_	*F*_000_ = 592
*M**_r_* = 278.35	*D*_x_ = 1.252 Mg m^−^^3^
Monoclinic, *P*2_1_/*a*	Mo *K*α radiation λ = 0.71070 Å
Hall symbol: -P 2yab	Cell parameters from 2169 reflections
*a* = 13.694 (13) Å	θ = 2.6–27.9º
*b* = 6.888 (6) Å	µ = 0.08 mm^−^^1^
*c* = 16.929 (16) Å	*T* = 293 (2) K
β = 112.417 (12)º	Platelet, colorless
*V* = 1476 (2) Å^3^	0.24 × 0.18 × 0.10 mm
*Z* = 4	

### Data collection

**Table:**

Rigaku Saturn diffractometer	2593 independent reflections
Radiation source: rotating anode	2004 reflections with *I* > 2σ(*I*)
Monochromator: confocal	*R*_int_ = 0.045
Detector resolution: 7.31 pixels mm^-1^	θ_max_ = 25.0º
*T* = 293(2) K	θ_min_ = 1.3º
ω scans	*h* = −16→16
Absorption correction: multi-scan(Jacobson, 1998)	*k* = −8→8
*T*_min_ = 0.982, *T*_max_ = 0.992	*l* = −20→20
10708 measured reflections	

### Refinement

**Table:**

Refinement on *F*^2^	Secondary atom site location: difference Fourier map
Least-squares matrix: full	Hydrogen site location: inferred from neighbouring sites
*R*[*F*^2^ > 2σ(*F*^2^)] = 0.064	H-atom parameters constrained
*wR*(*F*^2^) = 0.162	*w* = 1/[σ^2^(*F*_o_^2^) + (0.0737*P*)^2^ + 0.1595*P*] where *P* = (*F*_o_^2^ + 2*F*_c_^2^)/3
*S* = 1.13	(Δ/σ)_max_ = 0.001
2593 reflections	Δρ_max_ = 0.13 e Å^−^^3^
190 parameters	Δρ_min_ = −0.16 e Å^−^^3^
Primary atom site location: structure-invariant direct methods	Extinction correction: none

### Special details

**Table:**

Geometry. All esds (except the esd in the dihedral angle between two l.s. planes) are estimated using the full covariance matrix. The cell esds are taken into account individually in the estimation of esds in distances, angles and torsion angles; correlations between esds in cell parameters are only used when they are defined by crystal symmetry. An approximate (isotropic) treatment of cell esds is used for estimating esds involving l.s. planes.
Refinement. Refinement of *F*^2^ against ALL reflections. The weighted *R*-factor *wR* and goodness of fit *S* are based on *F*^2^, conventional *R*-factors *R* are based on *F*, with *F* set to zero for negative *F*^2^. The threshold expression of *F*^2^ > σ(*F*^2^) is used only for calculating *R*-factors(gt) *etc*. and is not relevant to the choice of reflections for refinement. *R*-factors based on *F*^2^ are statistically about twice as large as those based on *F*, and *R*- factors based on ALL data will be even larger.

### Fractional atomic coordinates and isotropic or equivalent isotropic displacement parameters (Å^2^)

**Table:**

	*x*	*y*	*z*	*U*_iso_*/*U*_eq_	
N1	0.22712 (17)	−0.0370 (3)	0.14030 (12)	0.0759 (7)	
H1A	0.2063	−0.1288	0.1645	0.091*	
H1B	0.2046	−0.0315	0.0855	0.091*	
N2	0.43578 (14)	0.4015 (3)	0.28441 (11)	0.0555 (5)	
N3	0.41678 (14)	0.5193 (3)	0.14393 (11)	0.0561 (5)	
N4	0.36441 (16)	0.4614 (3)	0.06027 (11)	0.0659 (6)	
C1	0.29571 (17)	0.0997 (3)	0.18869 (13)	0.0520 (6)	
C2	0.32949 (16)	0.2498 (3)	0.14847 (13)	0.0506 (6)	
C3	0.39672 (16)	0.3917 (3)	0.19859 (13)	0.0497 (5)	
C4	0.40415 (17)	0.2541 (3)	0.32127 (13)	0.0536 (6)	
C5	0.33628 (16)	0.1021 (3)	0.27801 (13)	0.0517 (6)	
C6	0.31279 (19)	0.3022 (4)	0.06373 (14)	0.0633 (7)	
H6	0.2703	0.2325	0.0159	0.076*	
C7	0.47904 (17)	0.6919 (3)	0.16101 (15)	0.0553 (6)	
C8	0.5064 (2)	0.7724 (4)	0.09698 (17)	0.0709 (7)	
H8	0.4837	0.7154	0.0432	0.085*	
C9	0.5679 (2)	0.9391 (4)	0.1146 (2)	0.0841 (9)	
H9	0.5862	0.9938	0.0719	0.101*	
C10	0.6020 (2)	1.0244 (4)	0.1935 (2)	0.0856 (9)	
H10	0.6434	1.1359	0.2045	0.103*	
C11	0.5746 (2)	0.9436 (4)	0.25592 (19)	0.0801 (8)	
H11	0.5973	1.0013	0.3096	0.096*	
C12	0.51355 (18)	0.7772 (4)	0.24043 (16)	0.0650 (7)	
H12	0.4959	0.7232	0.2836	0.078*	
C13	0.4445 (2)	0.2642 (4)	0.41739 (14)	0.0737 (8)	
H13A	0.4970	0.3665	0.4371	0.088*	
H13B	0.4791	0.1426	0.4409	0.088*	
C14	0.3584 (2)	0.3027 (4)	0.45167 (17)	0.0882 (9)	
H14A	0.3908	0.3592	0.5082	0.106*	
H14B	0.3100	0.3982	0.4152	0.106*	
C15	0.2955 (2)	0.1271 (5)	0.45736 (17)	0.0860 (9)	
H15A	0.2447	0.1677	0.4812	0.103*	
H15B	0.3433	0.0359	0.4971	0.103*	
C16	0.2365 (2)	0.0213 (4)	0.37396 (15)	0.0755 (8)	
H16A	0.1836	0.1081	0.3359	0.091*	
H16B	0.1997	−0.0886	0.3856	0.091*	
C17	0.3059 (2)	−0.0518 (4)	0.32817 (15)	0.0671 (7)	
H17A	0.3699	−0.1066	0.3702	0.081*	
H17B	0.2689	−0.1552	0.2894	0.081*	

### Atomic displacement parameters (Å^2^)

**Table:**

	*U*^11^	*U*^22^	*U*^33^	*U*^12^	*U*^13^	*U*^23^
N1	0.0947 (16)	0.0801 (15)	0.0543 (12)	−0.0340 (12)	0.0301 (12)	−0.0143 (10)
N2	0.0539 (11)	0.0634 (12)	0.0483 (11)	−0.0081 (9)	0.0185 (9)	−0.0006 (9)
N3	0.0578 (12)	0.0610 (12)	0.0471 (11)	−0.0037 (9)	0.0173 (9)	0.0050 (9)
N4	0.0722 (14)	0.0747 (14)	0.0465 (11)	−0.0064 (11)	0.0178 (10)	0.0053 (10)
C1	0.0523 (13)	0.0573 (13)	0.0484 (12)	−0.0041 (11)	0.0214 (11)	−0.0058 (10)
C2	0.0503 (13)	0.0581 (14)	0.0429 (12)	0.0013 (11)	0.0171 (10)	0.0002 (10)
C3	0.0476 (13)	0.0577 (13)	0.0447 (12)	−0.0002 (11)	0.0186 (10)	0.0035 (10)
C4	0.0493 (13)	0.0676 (15)	0.0439 (12)	−0.0047 (11)	0.0177 (10)	0.0006 (10)
C5	0.0530 (13)	0.0555 (13)	0.0485 (12)	−0.0017 (11)	0.0217 (10)	0.0010 (10)
C6	0.0683 (16)	0.0724 (16)	0.0447 (13)	−0.0090 (13)	0.0166 (11)	−0.0012 (11)
C7	0.0480 (13)	0.0531 (13)	0.0633 (14)	0.0038 (11)	0.0195 (11)	0.0097 (11)
C8	0.0694 (16)	0.0731 (17)	0.0712 (16)	0.0035 (14)	0.0278 (14)	0.0164 (13)
C9	0.081 (2)	0.0758 (19)	0.103 (2)	−0.0069 (16)	0.0428 (18)	0.0252 (17)
C10	0.081 (2)	0.0668 (18)	0.110 (2)	−0.0146 (15)	0.0371 (18)	0.0020 (17)
C11	0.0773 (19)	0.0689 (17)	0.096 (2)	−0.0126 (14)	0.0348 (16)	−0.0040 (15)
C12	0.0638 (16)	0.0633 (16)	0.0712 (16)	−0.0039 (12)	0.0294 (13)	0.0000 (12)
C13	0.0709 (17)	0.098 (2)	0.0462 (13)	−0.0243 (15)	0.0163 (12)	−0.0020 (13)
C14	0.111 (2)	0.102 (2)	0.0664 (17)	−0.0358 (18)	0.0502 (17)	−0.0252 (15)
C15	0.098 (2)	0.106 (2)	0.0694 (17)	−0.0220 (18)	0.0490 (17)	−0.0144 (15)
C16	0.0798 (18)	0.0903 (19)	0.0643 (16)	−0.0226 (15)	0.0364 (14)	−0.0022 (14)
C17	0.0829 (18)	0.0652 (16)	0.0568 (14)	−0.0123 (13)	0.0305 (13)	−0.0004 (12)

### Geometric parameters (Å, °)

**Table:**

N1—C1	1.361 (3)	C9—C10	1.368 (4)
N1—H1A	0.8600	C9—H9	0.9300
N1—H1B	0.8600	C10—C11	1.369 (4)
N2—C3	1.345 (3)	C10—H10	0.9300
N2—C4	1.347 (3)	C11—C12	1.383 (3)
N3—C3	1.377 (3)	C11—H11	0.9300
N3—N4	1.380 (3)	C12—H12	0.9300
N3—C7	1.427 (3)	C13—C14	1.523 (4)
N4—C6	1.318 (3)	C13—H13A	0.9700
C1—C5	1.398 (3)	C13—H13B	0.9700
C1—C2	1.410 (3)	C14—C15	1.508 (4)
C2—C3	1.388 (3)	C14—H14A	0.9700
C2—C6	1.411 (3)	C14—H14B	0.9700
C4—C5	1.406 (3)	C15—C16	1.519 (4)
C4—C13	1.507 (3)	C15—H15A	0.9700
C5—C17	1.512 (3)	C15—H15B	0.9700
C6—H6	0.9300	C16—C17	1.523 (3)
C7—C12	1.375 (3)	C16—H16A	0.9700
C7—C8	1.390 (3)	C16—H16B	0.9700
C8—C9	1.388 (4)	C17—H17A	0.9700
C8—H8	0.9300	C17—H17B	0.9700
			
C1—N1—H1A	120.0	C11—C10—H10	120.4
C1—N1—H1B	120.0	C10—C11—C12	121.0 (3)
H1A—N1—H1B	120.0	C10—C11—H11	119.5
C3—N2—C4	113.38 (18)	C12—C11—H11	119.5
C3—N3—N4	110.27 (19)	C7—C12—C11	119.8 (2)
C3—N3—C7	130.71 (19)	C7—C12—H12	120.1
N4—N3—C7	119.02 (18)	C11—C12—H12	120.1
C6—N4—N3	105.81 (18)	C4—C13—C14	113.6 (2)
N1—C1—C5	123.9 (2)	C4—C13—H13A	108.8
N1—C1—C2	119.7 (2)	C14—C13—H13A	108.8
C5—C1—C2	116.4 (2)	C4—C13—H13B	108.8
C3—C2—C1	118.98 (19)	C14—C13—H13B	108.8
C3—C2—C6	104.73 (19)	H13A—C13—H13B	107.7
C1—C2—C6	136.3 (2)	C15—C14—C13	115.4 (2)
N2—C3—N3	126.5 (2)	C15—C14—H14A	108.4
N2—C3—C2	126.41 (19)	C13—C14—H14A	108.4
N3—C3—C2	107.11 (19)	C15—C14—H14B	108.4
N2—C4—C5	125.8 (2)	C13—C14—H14B	108.4
N2—C4—C13	114.5 (2)	H14A—C14—H14B	107.5
C5—C4—C13	119.6 (2)	C14—C15—C16	116.1 (2)
C1—C5—C4	118.92 (19)	C14—C15—H15A	108.3
C1—C5—C17	121.2 (2)	C16—C15—H15A	108.3
C4—C5—C17	119.9 (2)	C14—C15—H15B	108.3
N4—C6—C2	112.1 (2)	C16—C15—H15B	108.3
N4—C6—H6	124.0	H15A—C15—H15B	107.4
C2—C6—H6	124.0	C15—C16—C17	114.7 (2)
C12—C7—C8	119.8 (2)	C15—C16—H16A	108.6
C12—C7—N3	120.7 (2)	C17—C16—H16A	108.6
C8—C7—N3	119.5 (2)	C15—C16—H16B	108.6
C9—C8—C7	119.0 (3)	C17—C16—H16B	108.6
C9—C8—H8	120.5	H16A—C16—H16B	107.6
C7—C8—H8	120.5	C5—C17—C16	114.4 (2)
C10—C9—C8	121.2 (3)	C5—C17—H17A	108.7
C10—C9—H9	119.4	C16—C17—H17A	108.7
C8—C9—H9	119.4	C5—C17—H17B	108.7
C9—C10—C11	119.2 (3)	C16—C17—H17B	108.7
C9—C10—H10	120.4	H17A—C17—H17B	107.6

### Hydrogen-bond geometry (Å, °)

**Table:**

*D*—H···*A*	*D*—H	H···*A*	*D*···*A*	*D*—H···*A*
N1—H1B···N4^i^	0.86	2.28	3.139 (4)	175
N1—H1A···Cg1^ii^	0.86	2.84	3.608 (4)	150

Symmetry codes: (i) −*x*+1/2, *y*−1/2, −*z*; (ii) *x*−3/2, −*y*−1/2, *z*.
